# Fast bias-corrected conductivity mapping using stimulated echoes

**DOI:** 10.1007/s10334-024-01194-3

**Published:** 2024-08-06

**Authors:** Santhosh Iyyakkunnel, Matthias Weigel, Oliver Bieri

**Affiliations:** 1 Division of Radiological Physics, Department of Radiology , University Hospital Basel, Basel, Switzerland; 2 Department of Biomedical Engineering, University of Basel, Basel, Switzerland; 3 Translational Imaging in Neurology (ThINk) Basel, Department of Biomedical Engineering, Faculty of Medicine, University Hospital Basel and University of Basel, Basel, Switzerland; 4 Neurologic Clinic and Policlinic, MS Center and Research Center for Clinical Neuroimmunology and Neuroscience Basel (RC2NB), University Hospital Basel and University of Basel, Basel, Switzerland

**Keywords:** Spin echo, Stimulated echo, B_1_ mapping, Electrical properties tomography, EPT, Conductivity

## Abstract

**Objective:**

To demonstrate the potential of a double angle stimulated echo (DA-STE) method for fast and accurate “full” homogeneous Helmholtz-based electrical properties tomography using a simultaneous ${B}_{1}^{+}$ magnitude and transceive phase measurement.

**Methods:**

The combination of a spin and stimulated echo can be used to yield an estimate of both ${B}_{1}^{+}$ magnitude and transceive phase and thus provides the means for “full" EPT reconstruction. An interleaved 2D acquisition scheme is used for rapid acquisition. The method was validated in a saline phantom and compared to a double angle method based on two single gradient echo acquisitions (GRE-DAM). The method was evaluated in the brain of a healthy volunteer.

**Results:**

The ${B}_{1}^{+}$ magnitude obtained with DA-STE showed excellent agreement with the GRE-DAM method. Conductivity values based on the “full” EPT reconstruction also agreed well with the expectations in the saline phantom. In the brain, the method delivered conductivity values close to literature values.

**Discussion:**

The method allows the use of the “full” Helmholtz-based EPT reconstruction without the need for additional measurements. As a result, quantitative conductivity values are improved compared to phase-based EPT reconstructions. DA-STE is a fast complex-${B}_{1}^{+}$ mapping technique that could render EPT clinically relevant at 3 T.

**Supplementary Information:**

The online version contains supplementary material available at 10.1007/s10334-024-01194-3.

## Introduction

Electrical properties tomography (EPT) facilitates the non-invasive retrieval of electrical tissue properties such as permittivity and conductivity with conventional MRI [1–3]. As a result, EPT gained some interest in the past years as a potentially new biomarker in cancer diagnosis and treatment planning [4–8]. Tissue conductivity is mainly a function of water content and ion concentration and was shown to increase in case of pathology [4, 6–8].

Electrical properties (EPs) are related to the radio frequency (RF) field used for excitation via the Maxwell equations [2, 9]. This complex-valued RF excitation field, commonly called ${B}_{1}^{+}$, is assessed based on the measurable estimates of its magnitude ($\left|{B}_{1}^{+}\right|$) and phase (${\varphi }^{+}=\angle {B}_{1}^{+}$). To be exact, the latter cannot be measured directly but is often approximated by the so-called transceive phase, ${\varphi }^{\pm }$ [10, 11]. Often the EPs are obtained under the assumption that the EPs are constant across one tissue type. Maxwell’s equations can then be rearranged to yield the EPs via the homogeneous Helmholtz equation [3, 11, 12]. The reconstruction of the EPs ideally requires knowledge of the complex ${B}_{1}^{+}$ field ($\left|{B}_{1}^{+}\right|{e}^{i{\varphi }^{+}}$), but simplified relations exist that allow a separate estimation of the permittivity or conductivity alone based on the RF field’s magnitude or phase, respectively [3, 11]. Phase-based EPT, i.e. disregarding $\left|{B}_{1}^{+}\right|$, however, usually results in an overestimation of the conductivity, especially at 3 T [3, 10, 11]. Even though a few MRI methods exist that yield simultaneous information of the ${B}_{1}^{+}$ magnitude and its phase [13–15], the components of the ${B}_{1}^{+}$ field are commonly obtained by two separate scans [10, 11, 16]. Most EPT studies focus on conductivity-only mapping using phase-based EPT [4, 7] accepting the resulting bias in favor of shorter scan times as the ${B}_{1}^{+}$ magnitude is not needed. For transceive phase measurements often a spin-echo sequence is used as it combines high signal with robustness against field inhomogeneities [12, 17, 18].

In this study, we show that a stimulated echo sequence with a double angle configuration (referred to as ‘DA-STE’ for the remainder of this article), first described by Akoka et al*.* [19], is very well suited for EPT and for conductivity mapping in specific. The suggested sequence generates both a spin and a stimulated echo, from which the simultaneous determination of the (transceive) ${B}_{1}^{+}$ phase and the ${B}_{1}^{+}$ magnitude is possible. The latter does not provide sufficient signal-to-noise for high-resolution permittivity mapping, but we will show that it is ideal for bias correction in conductivity mapping. Hence, no additional time is needed for full homogeneous Helmholtz-based EPT. The proposed method is directly compared to the double angle method with gradient echoes [20, 21] in phantom measurements. Furthermore, it is shown for in vivo measurements that DA-STE can deliver accurate, bias-corrected conductivity maps.

## Methods

### The double angle stimulated echo (DA-STE) sequence

**Fig. 1:**
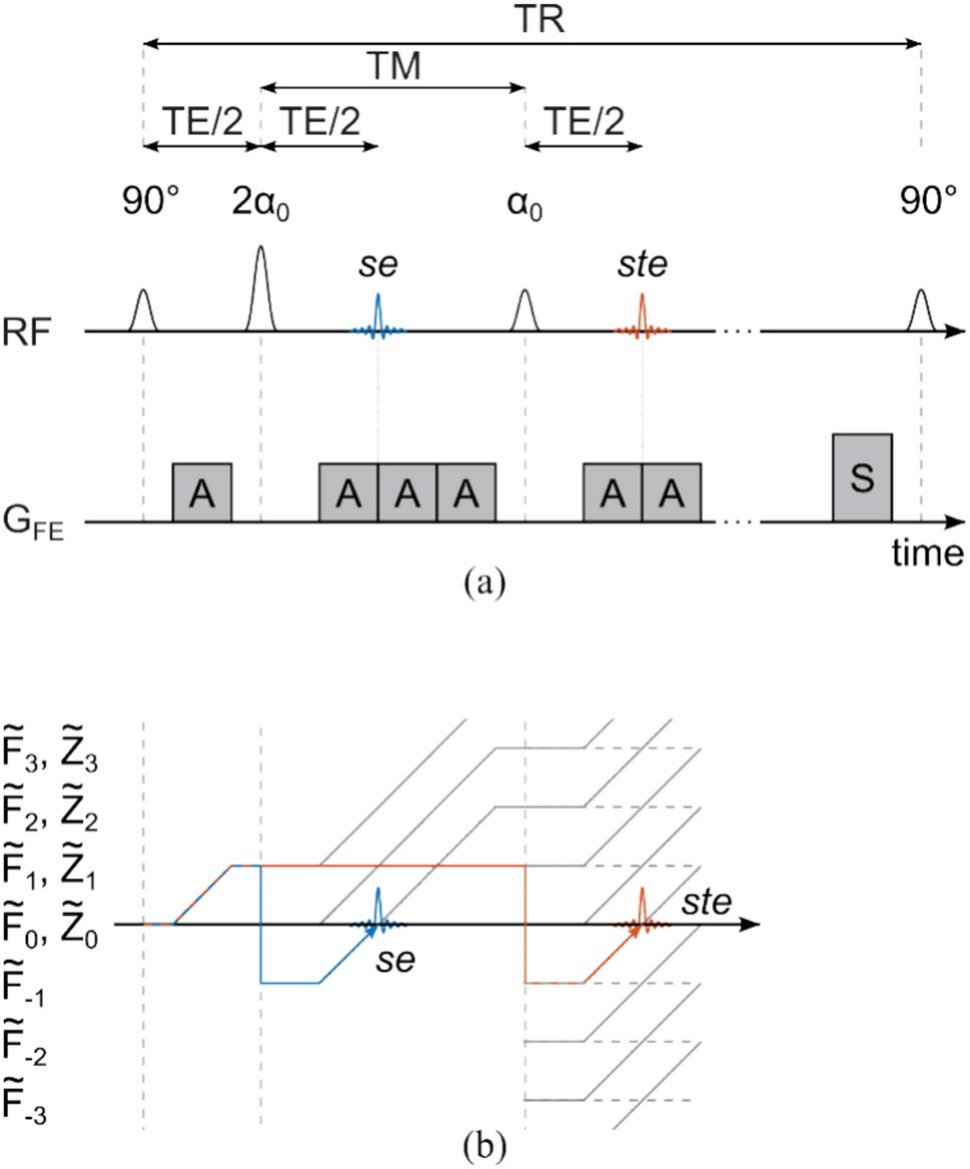
**a** simplified sequence diagram of the suggested DA-STE variant. **b** Schematic extended phase graph of the sequence in (**a**)

The suggested double angle stimulated echo (DA-STE) sequence including the relevant gradient moments is shown in Fig. 1(a). An extended phase graph [22, 23] diagram is shown in Fig. 1(b) to visualize the spin and stimulated echo formation. Generally, three RF pulses will generate up to four spin echoes and one stimulated echo depending on distance and dephasing [22, 23]. If the refocusing flip angle is not exactly 180°, the magnetization dephased by the first gradient moment will partly be ‘stored’ as modulated longitudinal magnetization [22, 23]. Longitudinal magnetization stays unaffected by gradient moments but relaxes with the time constant T_1_. The third RF pulse flips the modulated longitudinal magnetization back into the transverse plane to form a stimulated echo [22, 23]. In order to ensure proper separation of the stimulated echo path from the spin echo path, the spin echo is dephased by twice the dephasing gradient moment from the first period [24]. This is emphasized in the extended phase graph schematic in Fig. 1(b).

The signal intensities of the pure spin echo (${S}_{se}$) and the isolated stimulated echo (${S}_{ste}$) in a double angle configuration with an initial excitation pulse of 90° (as shown in Fig. 1(a)) are given as follows [19, 22–25]:

1 $$S_{se} = M_{0} \sin \left( {\text{B}_{1} \cdot \frac{\pi }{2}} \right)\sin^{2} \left( {\text{B}_{1} \cdot \alpha_{0} } \right)e^{{ - TE/T_{2} }}$$

2 $$S_{ste} = \frac{{M_{0} }}{2}\sin \left( {\text{B}_{1} \cdot \frac{\pi }{2}} \right)\sin \left( {\text{B}_{1} \cdot 2\alpha_{0} } \right)\sin \left( {\text{B}_{1} \cdot \alpha_{0} } \right)e^{{ - TE/T_{2} }} e^{{ - TM/T_{1} }}$$

In Equations [1] and [2], the magnitude ${M}_{0}$ is proportional to the proton density. In case of incomplete magnetization recovery, the steady state longitudinal magnetization before excitation can be used instead [26]. TM and TE are the mixing time and the echo time, respectively (see Fig. 1(a)). T_1_ is the longitudinal relaxation time and ${\alpha }_{0}$ refers to the nominal flip angle (as indicated in Fig. 1(a)). Conveniently, B_1_ refers to the relative $\left|{B}_{1}^{+}\right|$ which is calculated by the ratio of the actual and nominal flip angle ($\alpha /{\alpha }_{0}$). When TM << T_1_, the cosine of the echo intensity ratio directly yields an estimate of the local flip angle $\alpha$ [19, 27]:

3 $$\alpha = \text{B}_{1} \cdot \alpha_{0} \approx \cos^{ - 1} \left( {\frac{{S_{ste} }}{{S_{se} }}} \right)$$

Thus, the sequence provides an estimate of the local flip angle along with the phase from the pure spin echo SE. It is worth noting that Equation [3] is independent of the flip angle of the first excitation pulse as it cancels out in the signal ratio ${S}_{ste}/{S}_{se}$. Setting $\alpha$ to 90° (as shown in Fig. 1(a)) proves to be beneficial for the signal-to-noise ratio (SNR) for the transceive phase estimation (using both ${S}_{se}$) and for the estimation of the local B_1_ map (as can be verified with Monte Carlo simulations). Note that in case of a non-uniform slice profile, a bias is introduced to Equation [3] that needs to be accounted for to obtain the actual B_1_. Further details are given in section “Simulations”.

The uncertainty of various B_1_-mapping methods, such as DA-STE among others, has been investigated in-depth by Pohmann and Scheffler [26]. In the frame of its impact on EPT, the proposed method is in addition compared to the robust but lengthy double angle method using two gradient echo acquisitions [21] (GRE-DAM). The uncertainty was estimated by Monte Carlo simulations and analytically by error propagation according to Pohmann and Scheffler [26]. More detailed information is found in the Supplementary Information.

The uncertainty of the spin echo phase was estimated by the inverse of the SNR of the spin echo magnitude image [28]. Due to the averaging of two phase measurements with opposite read out gradient polarity, the uncertainty of the transceive phase is given by ${SD}_{{\varphi }^{\pm }}=\sqrt{2}\cdot {SN{R}_{Magn}}^{-1}$, where $SN{R}_{Magn}$ is the SNR of one of the two magnitude images. $SN{R}_{Magn}$ was obtained by the ratio of the average signal magnitude within the object and the standard deviation of the real part of the image in the background (air).

### Electrical property reconstruction

Under the assumption that the local EPs vary slowly, the conductivity $\sigma$ was reconstructed based on the homogeneous helmholtz (hH) equation [12, 29] yielding:

4 $$\sigma = \frac{1}{{\mu_{0} \omega }}{\text{Im}} \left\{ {\frac{{\nabla^{2} B_{1}^{ + } }}{{B_{1}^{ + } }}} \right\} = \frac{1}{{\mu_{0} \omega }}\left( {\nabla^{2} \varphi^{ + } + 2\frac{{\nabla \text{B}_{1} \cdot \nabla \varphi^{ + } }}{{\text{B}_{1} }}} \right)$$

where ${\mu }_{0}$ is the magnetic vacuum permeability and $\omega$ is the Larmor frequency. The conductivity $\sigma$ is mainly related to the leading term on the right side of Equation [4], which contains only the phase. To avoid additional scan time, the second term containing B_1_ is thus often neglected yielding the phase-based reconstruction formula [3, 11]:

5 $$\sigma \approx \frac{{\nabla^{2} \varphi^{ + } }}{{\mu_{0} \omega }}$$

The phase-based approximation can result in errors of 20% at locations of high B_1_ gradients in the brain at 3 T [11]. Therefore, for an accurate estimation of the tissue conductivity, the B_1_ information is needed (cf. Equation [4]). Because the transmit phase ${\varphi }^{+}$ is not directly accessible in MRI, it is approximated by half the measurable transceive phase ${\varphi }^{\pm }$ [10, 11]. The latter is the superposition of the transmit and the receive phase. These phases are assumed to be similar for symmetrical objects up to field strengths of 3 T if a quadrature birdcage coil-like configuration is used for transmission and reception. The full hH equation and the magnitude-based expression for the permittivity are given in the Supplementary Information (see Equations [S3] and [S4]).

The local Laplacian and gradients for each voxel were estimated by fitting a second-order polynomial in the voxel’s neighborhood [7]. To mitigate tissue boundary errors, fitting was only applied to voxels that have magnitude values within 25% of the center voxel [7, 12]. The Eps were subsequently smoothed with a tissue boundary preserving median filter [7, 12] using the same strategy. The window size for the Laplace estimation was set to 7 × 7 × 7 voxels and 13 × 13 × 13 voxels for the median filter. Skull stripping and was performed using the standard software package FSL (FMRIB Software Library v6.0, Oxford, United Kingdom) [30]. For segmentation, the TSE brain images were mapped to the standard MNI average brain atlas [31, 32] using FSL’s linear and non-linear registration tool [33–35]. The resulting white and gray matter probability masks [36] were registered back to the TSE images, which had the same position and resolution as the DA-STE images. Binary masks were then generated for probability values above 80% to estimate the average conductivity of white and gray matter.

### Imaging experiments

All scans were performed at 3 T (Magnetom Prisma, Siemens Healthineers, Erlangen, Germany) using a dual-tuned 1H/23Na quadrature head coil for transmission and reception (Rapid Biomedical, Rimpar, Germany). Acquisitions were performed both in a phantom and in the brain of a healthy volunteer. In vivo human experiments were approved by the local ethics committee and informed consent was obtained from the volunteer beforehand. The saline phantom contained a salt concentration of 3 g/L, resulting in a theoretically estimated conductivity of 0.5 S/m at the Larmor frequency and at room temperature [37]. Relaxation times were reduced to tissue-comparable relaxation times of T_1_ ~ 930 ms and T_2_ ~ 75 ms by adding 0.125 mM MnCl_2_.

The double angle method using two single gradient echo acquisitions [21] (GRE-DAM) is used as a reference method for the B_1_ map due to its similarity to the proposed method and its robustness to motion. For GRE-DAM, an estimate of the local flip angle $\alpha$ is obtained by $\alpha ={\text{cos}}^{-1}\left({S}_{2}/\left(2{S}_{1}\right)\right)$. ${S}_{1}$ and ${S}_{2}$ represent the signals of the gradient echo measurements with the flip angles ${\alpha }_{1}$ and ${\alpha }_{2}=2{\alpha }_{1}$, respectively. A long TR is used to ensure that the B_1_ map is largely free from T_1_ effects. The flip angle of the first and the second acquisition was set to 90° and 180°, respectively, to increase the SNR of the resulting B_1_ map.

The gold standard of the transceive phase is the average of two spin echo phase acquisitions with opposite readout gradient polarity and was used to verify that the phase from the first echo of the suggested DA-STE sequence indeed corresponds to the conventional spin echo phase and thus to the transceive phase.

The sequence parameters of the two reference methods (SE, GRE-DAM) and the suggested approach (DA-STE) are compiled in Table 1 for the phantom measurements. For DA-STE, the gradient moment A in Fig. 1(a) was set to induce a dephasing of 4π per voxel (~ twice the readout gradient moment) and RF spoiling was enabled for each slice. For the brain measurement, the field-of-view (FOV) was 192 × 168 × 120 mm^3^.

**Table 1:**
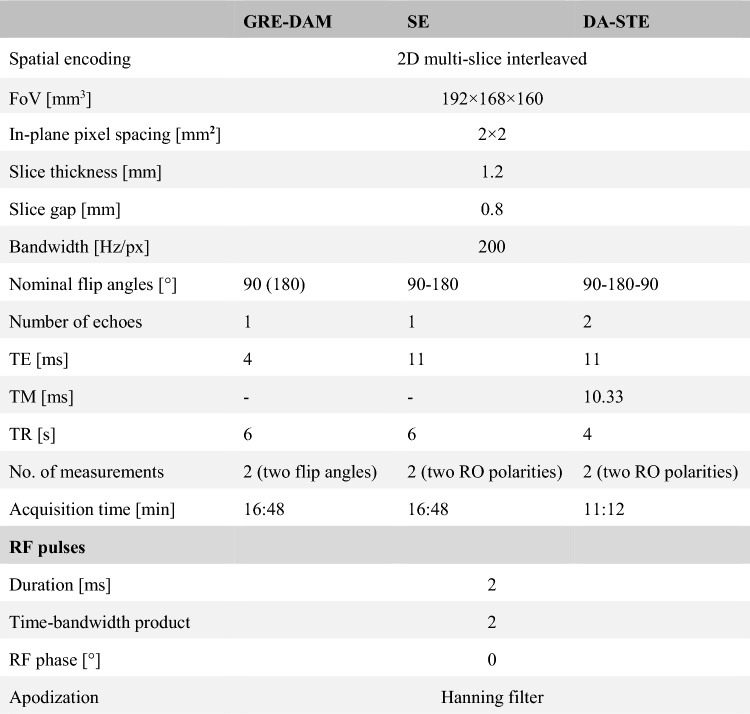
Sequence parameters for the GRE-DAM, the SE and the proposed DA-STE sequence. Different settings for separate experiments are given in brackets. GRE-DAM and the SE are the reference methods for the B1 map and the transceive phase, respectively. In the brain, the second GRE-DAM measurement used a TR of 7.86 s due to SAR restrictions

For tissue-boundary preserving estimation of the Laplacian and median filtering, a T_2_-weighted image was acquired with a 90°–180° turbo spin echo (TSE) sequence provided by the manufacturer of the system. The TR was set to 10 s with a TE of 105 ms and a receiver bandwidth of 200 Hz/px. The voxel size was 2 × 2 × 2 mm^3^ and scanning lasted 2:44 min.

### Simulations

All simulations and calculations were performed using MATLAB R2019a (The MathWorks, Natick, MA) unless stated otherwise.

For DA-STE, the ${S}_{se}$ and ${S}_{ste}$ signals were obtained using a 2D Bloch simulation for an ensemble of 361 × 361 magnetization vectors to simulate the effects within the xz-plane. The effects of the gradient moments in the frequency encoding (x) and slice selection (z) direction are thus accounted for individually. Signal simulation was performed using the parameter settings of Table 1. The impact of selective RF pulses was derived using the hard pulse approximation [38, 39].

Due to selective excitation, the flip angle estimated by Equation [3] must be corrected. To this end, ${S}_{se}$ and ${S}_{ste}$ signals of DA-STE were simulated for varying relative B_1_ values between 0.5 and 1.5 with a step size of 0.05. The resulting apparent flip angles (or rather B_1_) were calculated according to Equation [3] and correction was performed by interpolating the actual (input) B_1_ as a function of the apparent (output) B_1_, B_1,apparent_. For interpolation MATLAB’s interp1 function with the method ‘spline’ was used. Note that the correction was calculated for a fixed T_1_ and T_2_ (T_1_/T_2_ = 1 s/100 ms) which can result in a potential bias for deviating T_1_, such as for fluids. The same was done for the GRE-DAM for which T_1_ effects are smaller due to the longer TR.

## Results

**Fig. 2:**
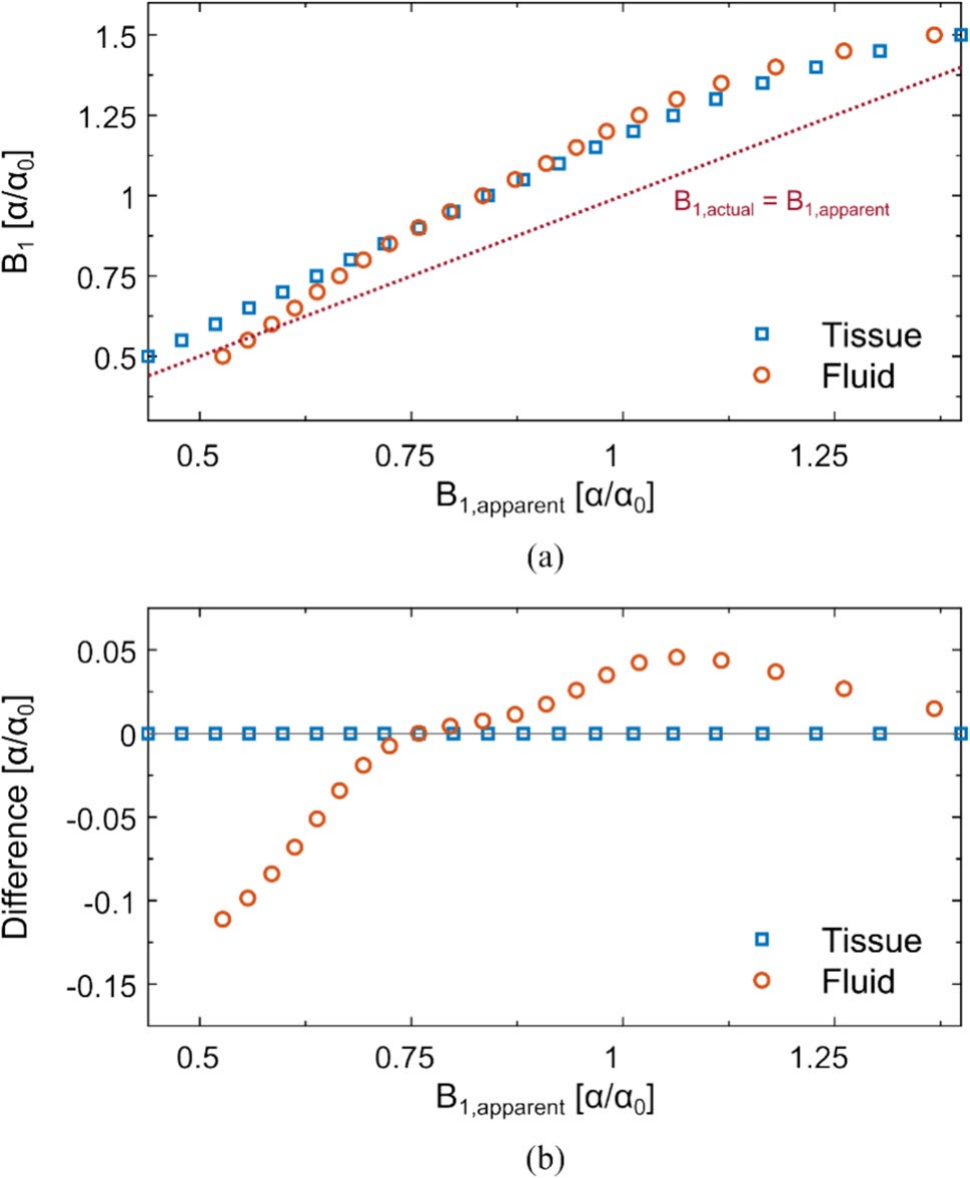
**a** Simulated results of the relationship between the apparent B_1_ due to the slice profile and the actual B_1_ using the proposed DA-STE sequence. The tissue data was obtained using relaxation times T_1_/T_2_ = 1 s/0.1 s. For Fluid, relaxation times were set to T_1_/T_2_ = 4 s/2 s. **b** Plot showing the difference in the actual B_1_ value (simulated) of fluid-like and tissue-like voxels with respect to the (interpolated) apparent B_1_

Simulations of the DA-STE signals, ${S}_{se}$ and ${S}_{ste}$, using the settings in Table 1 are used to find the relation between the resulting B_1,apparent_ and the actual B_1_ according to Equation [3]. Simulations were performed for relaxation times similar to brain tissue (T_1_/T_2_ = 1 s/0.1 s) and fluid (T_1_/T_2_ = 4 s/2 s) (see Fig. 2(a)). It is evident that B_1,apparent_ underestimates the actual B_1_ for most B_1_ values in the evaluated range. In Fig. 2(b), the difference between the ‘fluid’-like curve and the ‘tissue’-like curve is shown with respect to the apparent B_1_.

**Fig. 3:**
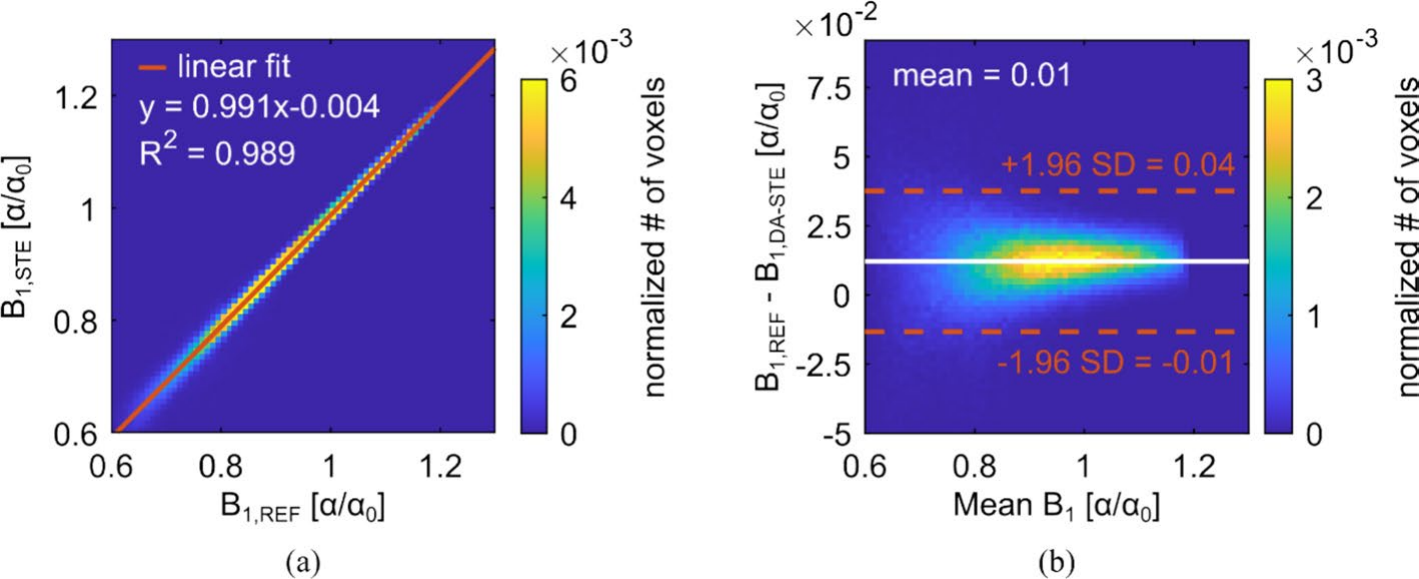
**a** Binned scatter plot showing the correlation between B_1,DA-STE_ and B_1,REF_. B_1,REF_ was obtained using the double angle method with two single gradient echo acquisitions (GRE-DAM). The orange line indicates the result of a linear least-squares analysis. **b** Bland–Altman plot of the two methods with lines marking the average difference and the 95% confidence intervals

For validation purposes, a direct voxel-wise comparison of the B_1_ maps obtained with the GRE-DAM (B_1,REF_) and the DA-STE (B_1,DA-STE_) method is shown in Fig. 3(a). Linear regression indicates a good fit of B_1,DA-STE_ with B_1,REF_. The parameters in relative units ($\alpha /{\alpha }_{0}$) of the fitted function yield a slope of 0.991 and an offset of -0.004. The Bland–Altman plot in Fig. 3(b) illustrates the difference of the B_1_ maps against the mean B_1_ value. The latter is defined as the voxel-wise mean B_1_ value of the two methods, (B_1,REF_ + B_1,DA-STE_)/2. The offset appears to be constant for different B_1_ values and thus indicates a global bias of 0.01 in relative units.

**Fig. 4:**
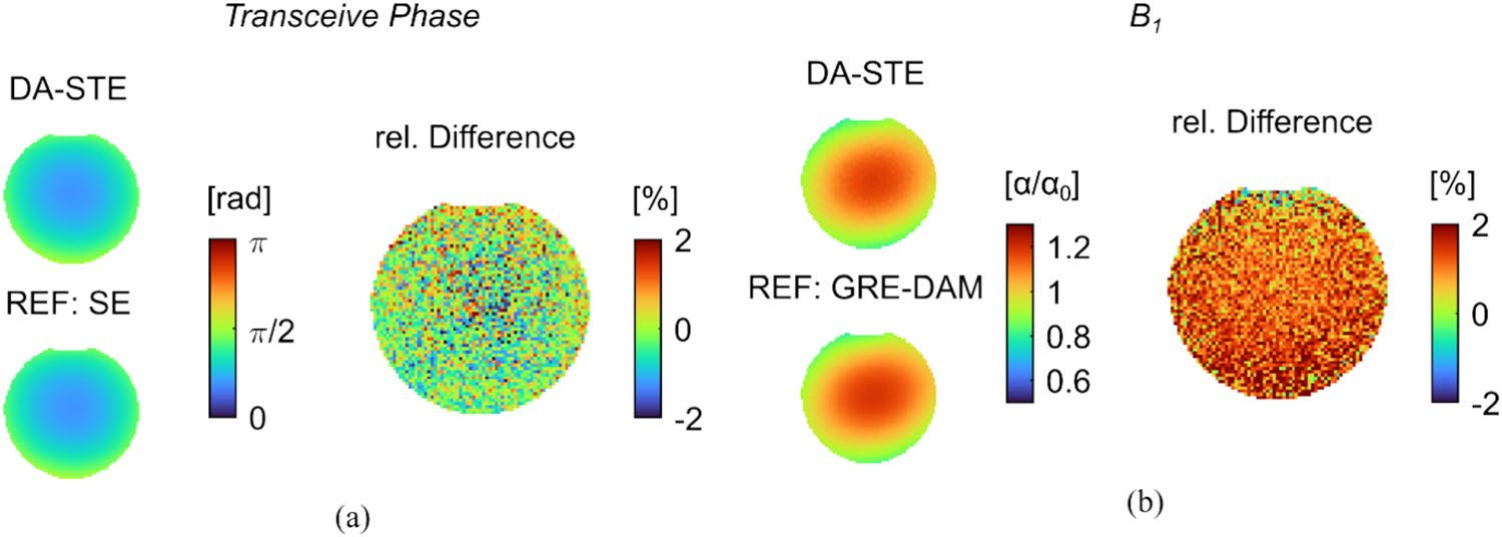
**a** Exemplary slice of the transceive maps obtained by the proposed DA-STE and the reference, a spin echo acquisition together with the relative difference in the same slice. **b** B_1_ maps obtained by the proposed DA-STE and the GRE-DAM reference method together with the relative difference in the same slice

The DA-STE method was compared to the conventional spin echo for the transceive phase and to the GRE-DAM for the B_1_ map. Exemplary slices of the transceive phase for the reference and the DA-STE are shown in Fig. 4(a). The shown transceive phase is the average of two separate phase measurements of opposite read out gradient polarities. The SNR of the corresponding magnitude images was measured to be around 160 for both the reference spin echo and the spin echo image of DA-STE. The uncertainty of the transceive phase was thus calculated to be 0.009 rad for both methods.

The GRE-DAM uncertainty was estimated using Monte Carlo simulations and had an average of around 0.002 ($\alpha /{\alpha }_{0}$) within the of B_1_ value range of [0.6, 1.3] ($\alpha /{\alpha }_{0}$). This is approximately three times less than the proposed DA-STE, which had an uncertainty of 0.006 ($\alpha /{\alpha }_{0}$) in the same B_1_ value range (see Supplementary Information Figure S1).

**Fig. 5:**
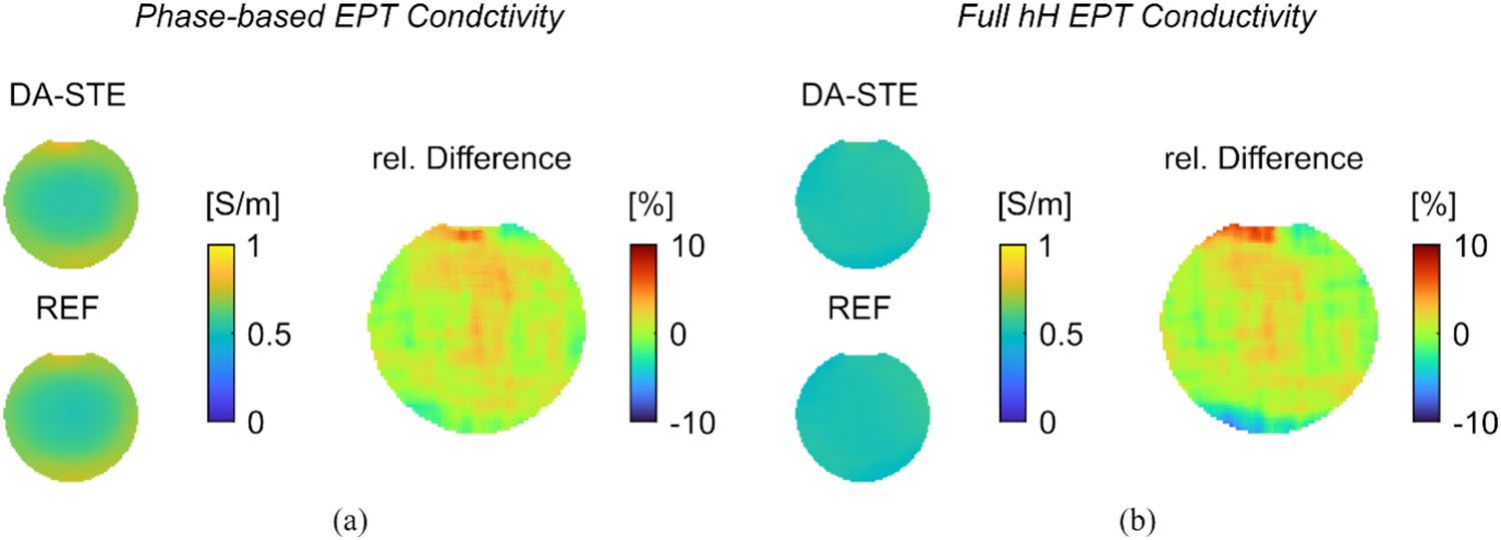
**a** Conductivity maps obtained by phase-based EPT for proposed DA-STE and the reference together with the relative difference in the same slice. **b** Conductivity maps obtained by full homogeneous Helmholtz EPT for the proposed DA-STE and the reference together with the relative difference in the same slice

Corresponding conductivity maps are presented in Fig. 5(a) and Fig. 5(b). The conductivities are shown once with the phase-based reconstruction (Equation [4], i.e., neglecting the B_1_ dependent term) in Fig. 5(a) and once with the full homogeneous Helmholtz equation (Equation [5], i.e., taking the B_1_ into account) in Fig. 5(b). The phased-based conductivity reconstruction exhibits a bowl shape for the homogeneous phantom [11]. Similar maps are obtained with the reference and the proposed method. Using the full homogeneous Helmholtz equation, the conductivity becomes more homogenous. For the reference method, the average conductivity over the whole phantom was found to be (0.51 ± 0.03) S/m. Due to outliers at the boundary, voxels within a width of 5 pixels from the phantom’s edge were excluded. For DA-STE, the average conductivity is also (0.51 ± 0.03) S/m. The average relative difference between the DA-STE and the reference conductivity was (0.03 ± 3.00) %. The corresponding permittivity maps reconstructed with the full homogeneous Helmholtz equation and the magnitude-based expression along with relative difference maps are shown in Supplementary Information Figure S4(a) and (b).

**Fig. 6:**
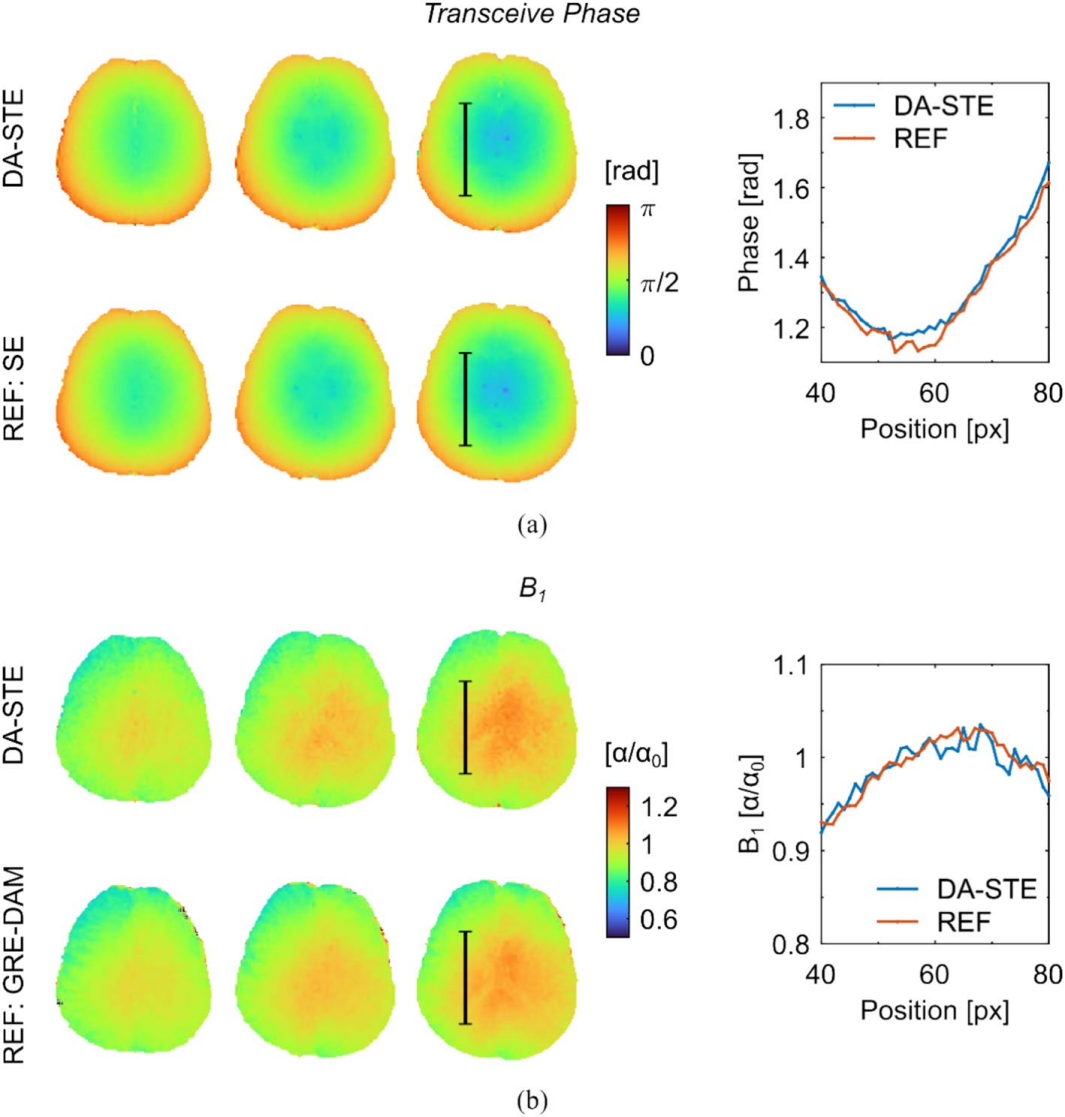
Three exemplary axial slices of the brain of a healthy volunteer. **a** Transceive phase maps of DA-STE (top row) and the reference (bottom row). Line profiles at the location indicated by the black line are shown on the right. **b** B_1_ maps of DA-STE (top row) and the reference (bottom row). Line profiles at the location indicated by the black line are shown on the right

Transceive phase and B_1_ maps of the in vivo brain experiment are compiled in Fig. 6. The figure shows the transceive phase and the B_1_ map for three different slices. The uncertainty of the transceive phase was approximately 0.02 rad for both DA-STE and the reference method. The uncertainty of the reference was only 4% lower. Line profiles in the brain tissue are also shown on the right of Fig. 6(a). The B_1_ maps in the brain estimated with DA-STE and the GRE-DAM are shown for the same slices in Fig. 6(b). Based on Monte Carlo simulations, the average uncertainty of the DA-STE B_1_ is estimated to be approximately 0.014 ($\alpha /{\alpha }_{0}$) in the brain tissue. The GRE-DAM B_1_ uncertainty is around 0.003 ($\alpha /{\alpha }_{0}$). Like for the transceive phase, line profiles of both, the DA-STE B_1_ and the reference B_1_, are shown. The corresponding brain slices of the TSE image acquired for the tissue boundary preserving Laplacian estimation and median filtering is shown in Supplementary Information Figure S3.

**Fig. 7:**
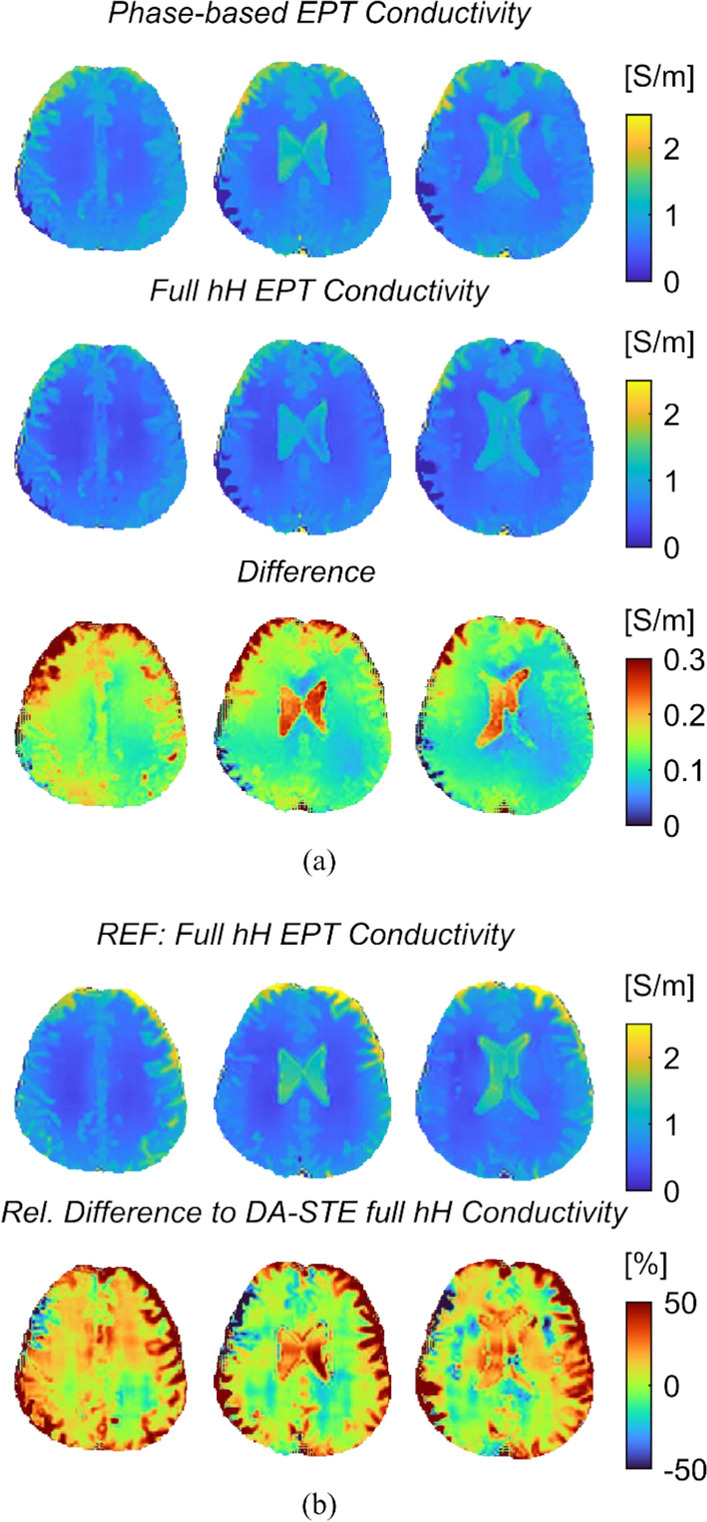
Conductivity reconstructions for the same axial slices shown in Fig. 6: **a** phase-based conductivity (top row), full hH conductivity (middle row) and the difference between the two (bottom row). **b** the full hH conductivity of the reference measurements and the relative difference to the full hH DA-STE conductivity

Using the transceive phase and the B_1_ map, conductivity maps were reconstructed for the same axial slices as in Fig. 6. Figure 7(a) shows the conductivity calculation based on the phase-based reconstruction (cf. Equation [5]), the conductivity based on the full equation (cf. Equation [4]) and the difference map between the two. Apart from the CSF, the bowl shape of the phase becomes evident in the difference map. In Fig. 7(b), the full hH EPT conductivity based on the reference measurements is shown alongside a map showing the relative difference between the reference conductivity and the DA-STE conductivity. For DA-STE, an average conductivity of 0.44 ± 0.06 S/m was found in white matter and 0.63 ± 0.12 S/m in gray matter. For the conductivity obtained with the reference methods, the values are 0.45 ± 0.08 S/m for white matter and 0.67 ± 0.12 S/m for gray matter. The permittivity results in the brain are shown in Supplementary Information Figure S5.

## Discussion

In the presented work, we investigated a double angle stimulated echo (DA-STE) method for its use in EPT. Due to the known difficulties of permittivity mapping at 3 T [16], all EPT reconstruction focused on conductivity mapping with the obtained B_1_ map for bias correction according to the full homogeneous Helmholtz reconstruction (Equation [4]). As a consequence, conductivity quantification in the human brain generally improved and yielded values that are closer to expectations.

The proposed method features a spin echo and thus allows for the direct measurement of the transceive phase. The local B_1_ information is obtained by the generation of only one additional echo, a stimulated echo. DA-STE is thus more time-efficient than the recently proposed Carr–Purcell multi-spin echo method for complex ${B}_{1}^{+}$ estimation [15]. To accomplish a stimulated echo free from other (overlapping) echo contributions, the dephasing of the spin echo signal needs to be large enough to avoid convergence of different pathways (see Fig. 1(b)) [24]. As stated by Akoka et al*.* [19], the mixing time TM should be minimized to avoid increased T_1_ relaxation effects leading to deviations from Equation [3].

Even when small mixing times are chosen, large T_1_ variation in the subject can bias B_1_ estimation. Even though the steady state longitudinal magnetization cancels out in the ratio of the echo signals (cf. Equation [3]), simulations showed that a residual T_1_ sensitivity pertains for short TR. This is most likely caused by remnant modulated longitudinal magnetization [40]. For most relaxation times of tissues, the deviation is small and negligible as the phantom measurements show, especially for long TR.

A 3D DA-STE sequence would contain considerable dead time and lead to unpractical scan times. A 2D multi-slice acquisition scheme, thus, makes efficient use of this mandatory long TR. However, due to the slice-selective pulses and the resulting flip angle distribution across the slice, the apparent B_1_ needs to be corrected to find the true B_1_. As shown in Fig. 2, the correction of the B_1_ depends on the relaxation times, especially on T_1_. While assuming one fixed T_1_ and T_2_ similar to brain tissues leads to negligible effects for different brain tissue types, larger deviations from the true B_1_ are expected in tissues or regions with a long T_1_ such as in CSF. The size of this T_1_-related bias on the conductivity is shown in the Supplementary Information Figure S2. It can be seen, that even for large differences in relaxation times, a bias in the conductivity is expected only in areas with large B_1_ gradients, specifically at lower B_1_ values. To avoid this, an additional correction fit for CSF using respective relaxation times can be used, for example.

A direct comparison of the DA-STE B_1_ map with the B_1_ map from the GRE-DAM sequence revealed very good agreement between the methods. A slight offset between the two B_1_ maps of 0.01 ($\alpha /{\alpha }_{0}$) is revealed in the phantom by the Bland–Altman Plot in Fig. 3(b) and can be observed in the relative difference map in Fig. 4(b). The small offset, however, is negligible for conductivity calculation and conductivity reconstruction according to the full homogeneous Helmholtz equation (Equation [4]). Conductivity reconstructions yielded values that are very close to expectations (0.5 S/m) with both methods in the phantom. With the focus on conductivity mapping, DA-STE profits from a high SNR transceive phase measurement at the cost of local B_1_ precision. The B_1_ containing term in the conductivity reconstruction formula (Equation [4]) can be regarded as a correction term that corrects for deviations at the periphery leading to the well-known bowl shape for phase-only reconstructions. The phantom’s permittivity reconstruction distinctly reveals the “anti-bowl” shape when the magnitude-only reconstruction is used (cf. Supplementary Information Figure S4). Like the conductivity, the permittivity becomes more homogeneous when using the full hH EPT reconstruction. In the brain, permittivity mapping proves challenging at 3 T due to higher B_1_ uncertainty, resulting in a strongly inhomogeneous permittivity map [16]. Nevertheless, permittivity values are in a reasonable range, supporting the presented method (cf. Supplementary Information Figure S5).

With the presented method, an overall improvement of the conductivity values could be observed, in the phantom as well as in the brain. The tissue conductivities in the human brain reported in literature [41, 42] are 0.34 S/m for WM and 0.59 S/m for GM and 2.14 S/m for CSF. The reconstructed average conductivities in white and gray matter (WM: 0.44 ± 0.06 S/m; GM: 0.63 ± 0.12 S/m) aligns well with literature values. The accuracy and precision in CSF is generally challenging because of the use of large kernels, partial volume effects and relaxation times in addition to cardiac pulsation [43]. For DA-STE, the B_1_ accuracy in CSF is additionally lowered when the adjustment of the local B_1_ is not extended to large T_1_ values.

In clinical practice, generally multichannel receive coils are used. However, to comply with the EPT model assumptions, the measurements were carried out with a birdcage coil and thus could not benefit from parallel acquisition techniques such as GRAPPA [44]. For EPT, accuracy of the transceive phase is crucial and thus the use of a multichannel reception coil requires a coil combination technique that ensures correct reconstruction of the transceive phase, such as for example the Roemer/SENSE approach [45, 46]. In particular for phase-based EPT, i.e. neglecting the B_1_ curvature, a coil combination technique was proposed by Lee et al*.* [47]. In the case of optimal coil combination, the acquisition time of the proposed sequence can be significantly reduced by enabling parallel acquisition techniques, making it a fast and accurate conductivity mapping method.

Compared to other methods that simultaneously measure the B_1_ magnitude and phase, such as multi-echo actual flip angle imaging (AFI) [13] and the recently proposed Carr–Purcell (CP) spin echo sequence [15], DA-STE offers distinct advantages. AFI is known to be prone to artifacts in vivo that stem mainly from incomplete spoiling of fat magnetization and thus needs adjustment [48]. Being spin echo based, DA-STE is robust against chemical shifts and field inhomogeneities. The CP sequence extracts the local flip angle information from the signal modulation across multiple echoes. Therefore, a long echo train is needed that ultimately leads to longer scan times for the same resolution and number of slices. Additionally, in the CP sequence, the refocusing pulses are typically around 90°, resulting in a transceive phase uncertainty that is roughly doubled to that of the proposed DA-STE transceive phase.

## Conclusion

A multi-slice dual spin echo and stimulated echo sequence offers rapid acquisition of the B_1_ and the transceive phase for B_1_-corrected conductivity mapping of the brain. In conclusion, the proposed method shows excellent prospects for bias-corrected conductivity mapping in the clinics.

## Supplementary Information

Below is the link to the electronic supplementary material.

Supplementary file1 (DOCX 2821 KB)

## Data Availability

The phantom measurements are available on reasonable request. Due to ethical considerations, the in vivo measurements are not publicly available.

## References

[1] Wen H (2003) Noninvasive quantitative mapping of conductivity and dielectric distributions using RF wave propagation effects in high-field MRI. In: Medical Imaging 2003: Physics of Medical Imaging. International Society for Optics and Photonics, pp 471–477

[2] Katscher U, Voigt T, Findeklee C, Vernickel P, Nehrke K, Doessel O (2009) Determination of electric conductivity and local SAR via B1 mapping. IEEE Trans Med Imaging 28:1365–1374. 10.1109/TMI.2009.2015757 19369153 10.1109/TMI.2009.2015757

[3] Voigt T, Katscher U, Doessel O (2011) Quantitative conductivity and permittivity imaging of the human brain using electric properties tomography. Magn Reson Med 66:456–466. 10.1002/mrm.22832 21773985 10.1002/mrm.22832

[4] Tha KK, Katscher U, Yamaguchi S, Stehning C, Terasaka S, Fujima N, Kudo K, Kazumata K, Yamamoto T, Van Cauteren M, Shirato H (2018) Noninvasive electrical conductivity measurement by MRI: a test of its validity and the electrical conductivity characteristics of glioma. Eur Radiol 28:348–355. 10.1007/s00330-017-4942-5 28698943 10.1007/s00330-017-4942-5

[5] Shin J, Kim MJ, Lee J, Nam Y, Kim M, Choi N, Kim S, Kim D-H (2015) Initial study on in vivo conductivity mapping of breast cancer using MRI. J Magn Reson Imaging 42:371–378. 10.1002/jmri.24803 25413153 10.1002/jmri.24803

[6] Mori N, Tsuchiya K, Sheth D, Mugikura S, Takase K, Katscher U, Abe H (2019) Diagnostic value of electric properties tomography (EPT) for differentiating benign from malignant breast lesions: comparison with standard dynamic contrast-enhanced MRI. Eur Radiol 29:1778–1786. 10.1007/s00330-018-5708-4 30255252 10.1007/s00330-018-5708-4

[7] Katscher U, Djamshidi K, Voigt T, Ivancevic MK, Abe H, Newstead GM, Keupp J (2012) Estimation of breast tumor conductivity using parabolic phase fitting. In: Proceedings of the 20th Annual Meeting of ISMRM, Melbourne, Australia, p Abstract no. 3482

[8] Balidemaj E, de Boer P, van Lier ALHMW, Remis RF, Stalpers LJA, Westerveld GH, Nederveen AJ, van den Berg CAT, Crezee J (2016) In vivo electric conductivity of cervical cancer patients based on B1+ maps at 3T MRI. Phys Med Biol 61:1596–1607. 10.1088/0031-9155/61/4/1596 26836010 10.1088/0031-9155/61/4/1596

[9] Leijsen R, Brink W, van den Berg C, Webb A, Remis R (2021) Electrical properties tomography: a methodological review. Diagnostics 11:176. 10.3390/diagnostics11020176 33530587 10.3390/diagnostics11020176 PMC7910937

[10] van Lier ALHMW, Brunner DO, Pruessmann KP, Klomp DWJ, Luijten PR, Lagendijk JJW, van den Berg CAT (2012) B1+ Phase mapping at 7 T and its application for in vivo electrical conductivity mapping. Magn Reson Med 67:552–561. 10.1002/mrm.22995 21710613 10.1002/mrm.22995

[11] van Lier ALHMW, Raaijmakers A, Voigt T, Lagendijk JJW, Luijten PR, Katscher U, van den Berg CAT (2014) Electrical properties tomography in the human brain at 1.5, 3, and 7T: a comparison study. Magn Reson Med 71:354–363. 10.1002/mrm.24637 23401276 10.1002/mrm.24637

[12] Katscher U, van den Berg CAT (2017) Electric properties tomography: biochemical, physical and technical background, evaluation and clinical applications. NMR Biomed 30:e3729. 10.1002/nbm.3729 10.1002/nbm.3729 28543640

[13] Choi N, Lee J, Kim M-O, Shin J, Kim D-H (2014) A modified multi-echo AFI for simultaneous B1+ magnitude and phase mapping. Magn Reson Imaging 32:314–320. 10.1016/j.mri.2013.12.005 24512801 10.1016/j.mri.2013.12.005

[14] Nehrke K, Börnert P (2012) DREAM—a novel approach for robust, ultrafast, multislice B1 mapping. Magn Reson Med 68:1517–1526. 10.1002/mrm.24158 22252850 10.1002/mrm.24158

[15] Iyyakkunnel S, Weigel M, Ganter C, Bieri O (2022) Complex B1+ mapping with Carr-Purcell spin echoes and its application to electrical properties tomography. Magn Reson Med 87:1250–1260. 10.1002/mrm.29020 34752636 10.1002/mrm.29020 PMC9298742

[16] Gavazzi S, van den Berg CAT, Sbrizzi A, Kok HP, Stalpers LJA, Lagendijk JJW, Crezee H, van Lier ALHMW (2019) Accuracy and precision of electrical permittivity mapping at 3T: the impact of three mapping techniques. Magn Reson Med 81:3628–3642. 10.1002/mrm.27675 30737816 10.1002/mrm.27675 PMC6593818

[17] Gavazzi S, Shcherbakova Y, Bartels LW, Stalpers LJA, Lagendijk JJW, Crezee H, van den Berg CAT, van Lier ALHMW (2020) Transceive phase mapping using the PLANET method and its application for conductivity mapping in the brain. Magn Reson Med 83:590–607. 10.1002/mrm.27958 31483520 10.1002/mrm.27958 PMC6900152

[18] Mandija S, Petrov PI, Vink JJT, Neggers SFW, van den Berg CAT (2021) Brain tissue conductivity measurements with mr-electrical properties tomography: an in vivo study. Brain Topogr 34:56–63. 10.1007/s10548-020-00813-1 33289858 10.1007/s10548-020-00813-1 PMC7803705

[19] Akoka S, Franconi F, Seguin F, Le Pape A (1993) Radiofrequency map of an NMR coil by imaging. Magn Reson Imaging 11:437–441. 10.1016/0730-725X(93)90078-R 8505878 10.1016/0730-725x(93)90078-r

[20] Insko EK, Bolinger L (1993) Mapping of the radiofrequency field. J Magn Reson Ser A 103:82–85. 10.1006/jmra.1993.1133

[21] Stollberger R, Wach P (1996) Imaging of the active B1 field in vivo. Magn Reson Med 35:246–251. 10.1002/mrm.1910350217 8622590 10.1002/mrm.1910350217

[22] Hennig J (1991) Echoes—how to generate, recognize, use or avoid them in MR-imaging sequences. Part I: fundamental and not so fundamental properties of spin echoes. Concepts Magn Reson 3:125–143. 10.1002/cmr.1820030302

[23] Weigel M (2015) Extended phase graphs: dephasing, RF pulses, and echoes—pure and simple. J Magn Reson Imaging 41:266–295. 10.1002/jmri.24619 24737382 10.1002/jmri.24619

[24] Burstein D (1996) Stimulated echoes: description, applications, practical hints. Concepts Magn Reson 8:269–278. 10.1002/(SICI)1099-0534(1996)8:4%3c269::AID-CMR3%3e3.0.CO;2-X

[25] Hahn EL (1950) Spin echoes. Phys Rev 80:580–594. 10.1103/PhysRev.80.580

[26] Pohmann R, Scheffler K (2013) A theoretical and experimental comparison of different techniques for B1 mapping at very high fields. NMR Biomed 26:265–275. 10.1002/nbm.2844 22972684 10.1002/nbm.2844

[27] Jiru F, Klose U (2006) Fast 3D radiofrequency field mapping using echo-planar imaging. Magn Reson Med 56:1375–1379. 10.1002/mrm.21083 17089359 10.1002/mrm.21083

[28] Gudbjartsson H, Patz S (1995) The rician distribution of noisy mri data. Magn Reson Med 34:910–914. 10.1002/mrm.1910340618 8598820 10.1002/mrm.1910340618 PMC2254141

[29] Katscher U, Kim D-H, Seo JK (2013) Recent progress and future challenges in MR electric properties tomography. Comput Math Method M. 10.1155/2013/546562 10.1155/2013/546562 PMC3614062 23573170

[30] Woolrich MW, Jbabdi S, Patenaude B, Chappell M, Makni S, Behrens T, Beckmann C, Jenkinson M, Smith SM (2009) Bayesian analysis of neuroimaging data in FSL. Neuroimage 45:S173–S186. 10.1016/j.neuroimage.2008.10.055 19059349 10.1016/j.neuroimage.2008.10.055

[31] Fonov V, Evans AC, Botteron K, Almli CR, McKinstry RC, Collins DL (2011) Unbiased average age-appropriate atlases for pediatric studies. Neuroimage 54:313–327. 10.1016/j.neuroimage.2010.07.033 20656036 10.1016/j.neuroimage.2010.07.033 PMC2962759

[32] Fonov V, Evans A, McKinstry R, Almli C, Collins D (2009) Unbiased nonlinear average age-appropriate brain templates from birth to adulthood. Neuroimage 47:S102. 10.1016/S1053-8119(09)70884-5

[33] Jenkinson M, Smith S (2001) A global optimisation method for robust affine registration of brain images. Med Image Anal 5:143–156. 10.1016/S1361-8415(01)00036-6 11516708 10.1016/s1361-8415(01)00036-6

[34] Jenkinson M, Bannister P, Brady M, Smith S (2002) Improved optimization for the robust and accurate linear registration and motion correction of brain images. Neuroimage 17:825–841. 10.1006/nimg.2002.1132 12377157 10.1016/s1053-8119(02)91132-8

[35] Greve DN, Fischl B (2009) Accurate and robust brain image alignment using boundary-based registration. Neuroimage 48:63–72. 10.1016/j.neuroimage.2009.06.060 19573611 10.1016/j.neuroimage.2009.06.060 PMC2733527

[36] Collins DL, Zijdenbos AP, Baaré WFC, Evans AC (1999) ANIMAL+INSECT: improved cortical structure segmentation. In: Kuba A, Šáamal M, Todd-Pokropek A (eds) Information Processing in Medical Imaging. Springer, Berlin, pp 210–223

[37] Stogryn A (1971) Equations for calculating the dielectric constant of saline water (correspondence). IEEE Trans Microw Theory Tech 19:733–736. 10.1109/TMTT.1971.1127617

[38] Pauly J, Le Roux P, Nishimura D, Macovski A (1991) Parameter relations for the Shinnar-Le Roux selective excitation pulse design algorithm (NMR imaging). IEEE Trans Med Imaging 10:53–65. 10.1109/42.75611 18222800 10.1109/42.75611

[39] Subramanian V, Eleff S, Rehn S, Leigh J (1985) An exact synthesis procedure for frequency selective pulses. In: Proceedings of the 4th Annual Meeting of SMRM, Boston, Massachusetts, USA, pp 1452–1453

[40] Weigel M, Zaitsev M, Hennig J (2007) Inversion recovery prepared turbo spin echo sequences with reduced SAR using smooth transitions between pseudo steady states. Magn Reson Med 57:631–637. 10.1002/mrm.21170 17326168 10.1002/mrm.21170

[41] Gabriel S, Lau RW, Gabriel C (1996) The dielectric properties of biological tissues: II. Measurements in the frequency range 10 Hz to 20 GHz. Phys Med Biol 41:2251–2269. 10.1088/0031-9155/41/11/002 8938025 10.1088/0031-9155/41/11/002

[42] Gabriel S, Lau RW, Gabriel C (1996) The dielectric properties of biological tissues: III. Parametric models for the dielectric spectrum of tissues. Phys Med Biol 41:2271–2293. 10.1088/0031-9155/41/11/003 8938026 10.1088/0031-9155/41/11/003

[43] Katscher U, Stehning C, Tha KK (2018) The impact of CSF pulsation on reconstructed brain conductivity. In: Proceedings of the 27th Annual Meeting of ISMRM, Paris, France, p Abstract no. 0546

[44] Griswold MA, Jakob PM, Heidemann RM, Nittka M, Jellus V, Wang J, Kiefer B, Haase A (2002) Generalized autocalibrating partially parallel acquisitions (GRAPPA). Magn Reson Med 47:1202–1210. 10.1002/mrm.10171 12111967 10.1002/mrm.10171

[45] Pruessmann KP, Weiger M, Scheidegger MB, Boesiger P (1999) SENSE: sensitivity encoding for fast MRI. Magn Reson Med 42:952–962. 10.1002/(SICI)1522-2594(199911)42:5%3c952::AID-MRM16%3e3.0.CO;2-S 10542355

[46] Roemer PB, Edelstein WA, Hayes CE, Souza SP, Mueller OM (1990) The NMR phased array. Magn Reson Med 16:192–225. 10.1002/mrm.1910160203 2266841 10.1002/mrm.1910160203

[47] Lee J, Shin J, Kim D-H (2016) MR-based conductivity imaging using multiple receiver coils. Magn Reson Med 76:530–539. 10.1002/mrm.25891 26375762 10.1002/mrm.25891

[48] Samsonov AA, Yarnykh VL (2024) Accurate actual flip angle imaging (AFI) in the presence of fat. Magn Reson Medi. 10.1002/mrm.30000 10.1002/mrm.30000 PMC10997465 38193249

