# Supplementary material for: Fast bias-corrected conductivity mapping using stimulated echoes
Source: MAGMA. 2024 Aug 6;37(6):1047–57. doi: 10.1007/s10334-024-01194-3 (PMC11582100; doi:10.1007/s10334-024-01194-3)
Supplement: Supplementary file 1 — Supplementary file1 (DOCX 2821 KB) [file 10334_2024_1194_MOESM1_ESM.docx]

**Fast Bias-Corrected Conductivity Mapping using Stimulated Echoes**

***Supplementary Information***

Santhosh Iyyakkunnel^1,2,*^, Matthias Weigel^1,2,3,4^ and Oliver Bieri^1,2^

**Author Affiliations:**

*^1^ Division of Radiological Physics, Department of Medical Radiology, University Hospital Basel, Basel, Switzerland*

*^2^ Department of Biomedical Engineering, University of Basel, Basel, Switzerland*

*^3^ Translational Imaging in Neurology (ThINk) Basel, Department of Biomedical Engineering, Faculty of Medicine, University Hospital Basel and University of Basel, Basel, Switzerland*

*^4^ Neurologic Clinic and Policlinic, MS Center and Research Center for Clinical Neuroimmunology and Neuroscience Basel (RC2NB), University Hospital Basel and University of Basel, Basel, Switzerland*

**Uncertainty of the B1**

Monte Carlo (MC) simulations were used to estimate the uncertainty of the B_1_ for DA-STE and the GRE-DAM reference method. Since selective excitation leads to a non-uniform flip angle distribution, the slice profile was derived using the hard pulse approximation and gaussian noise was added to the flip angle dependent signals. The noise level was adapted to the SNR, as observed in the phantom (noise = $1/SNR_{S_{1}}$, where $SNR_{S_{1}}$ corresponds to the SNR of the first acquired image for the respective B_1_ method). The apparent flip angle (or B_1_) was calculated using the corresponding flip angle formulas for DA-STE and GRE-DAM, respectively. For the MC simulation, 10’000 runs were performed. Results for T_1_ / T_2_ = 1 s / 0.1 s are shown in Supplementary Information Figure S1.

For validation purposes, analytic results for a uniform flip angle distribution (3D acquisition) are also provided. For the proposed DA-STE, the flip angle uncertainty $u_{\alpha}$ based on the law of error propagation is given by [1]:

|  | $u_{\alpha, DA-STE}=\sqrt{\frac{S_{se}^{2}+S_{ste}^{2}}{S_{se}^{2}\cdot(S_{se}^{2}\cdot e^{-\frac{2\cdot TM}{T_{1}}}-S_{ste}^{2})}}$ | [S1] |
| --- | --- | --- |

Similarly, the expression for the uncertainty of the reference method (GRE-DAM) can be derived:

|  | $u_{\alpha, GRE-DAM}=\sqrt{\frac{S_{1}^{2}+S_{2}^{2}}{S_{1}^{2}\cdot(4\cdot S_{1}^{2}-S_{2}^{2})}}$ | [S2] |
| --- | --- | --- |


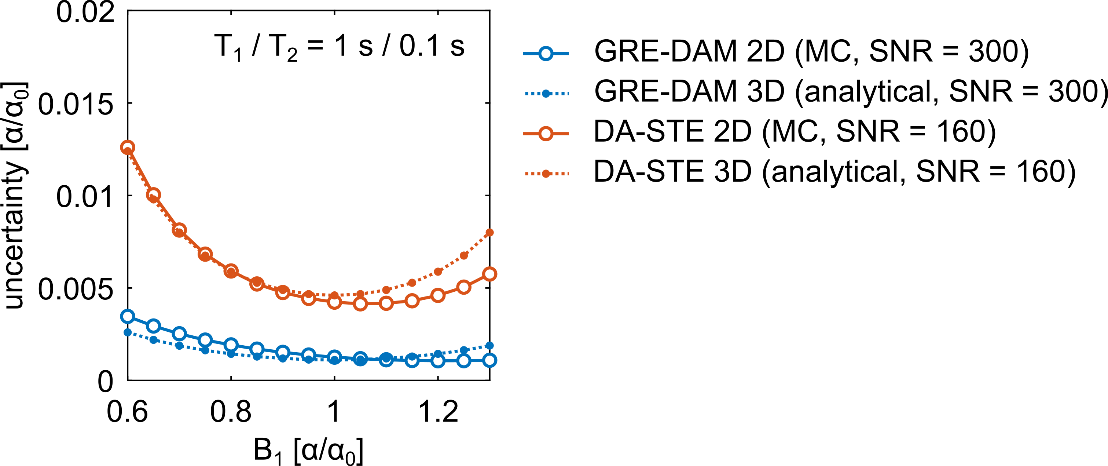


**Supplementary Information Figure S1:** Theoretical uncertainty of the DA-STE and the GRE-DAM B_1_ maps as a function of the B_1_ in relative units, ($\alpha/\alpha_{0}$). Analytical calculations are based on the law of error propagation (as in [1]) in case of a 3D acquisition, while MC simulations were used to account for selective excitation (2D). The relaxation times and the sequence parameters were chosen in accordance with the performed phantom measurements. The assumed SNR corresponds roughly to the estimated SNR in the acquired images.

**Potential bias in the conductivity due to relaxation related inaccuracies in B_1_**

For DA-STE, the estimated B_1_ assumes the same, global, T_1_ / T_2_ = 1 s / 0.1 s for all tissues (see Figure 2). Thus, a significant deviation from the presumed (fixed) relaxation times (e.g., for fluids) leads to inaccuracies in the estimated B_1_ that, in turn, may lead to a bias in the estimated conductivity. This effect is examined in the following for the phantom measurements (see Figure 4). For this purpose, the reference GRE-DAM B_1_ (see Figure 4b) is used to derive a hypothetical B_1,apparent_$\mathrm{for}$any given pair of T_1_ and T_2_ values. Following the methods as described in the ‘Simulations’ section, a B_1_ map that is now biased by the potential relaxation times mismatch is obtained. Together with the reference transceive phase (see Figure 4a), the full hH phantom conductivity has been calculated for the following cases:

1. T_1_ / T_2_ = 4 s / 2 s (fluid-like)
2. T_1_ / T_2_ = 2 s / 0.2 s (hypothetical pathology)
3. T_1_ / T_2_ = 930 ms / 75 ms (measured phantom)

The relative difference between the full hH reference conductivity and the three cases I – III, as well as to the phase-based conductivity, is shown in the Supplementary Information Figure S2. As expected,

B_1_ correction generally improves the estimation of the conductivity substantially compared to phase-based conductivity mapping. In the case of the full hH conductivity, a relaxation-related B_1_ error in the conductivity becomes noticeable only at low B_1_ values.


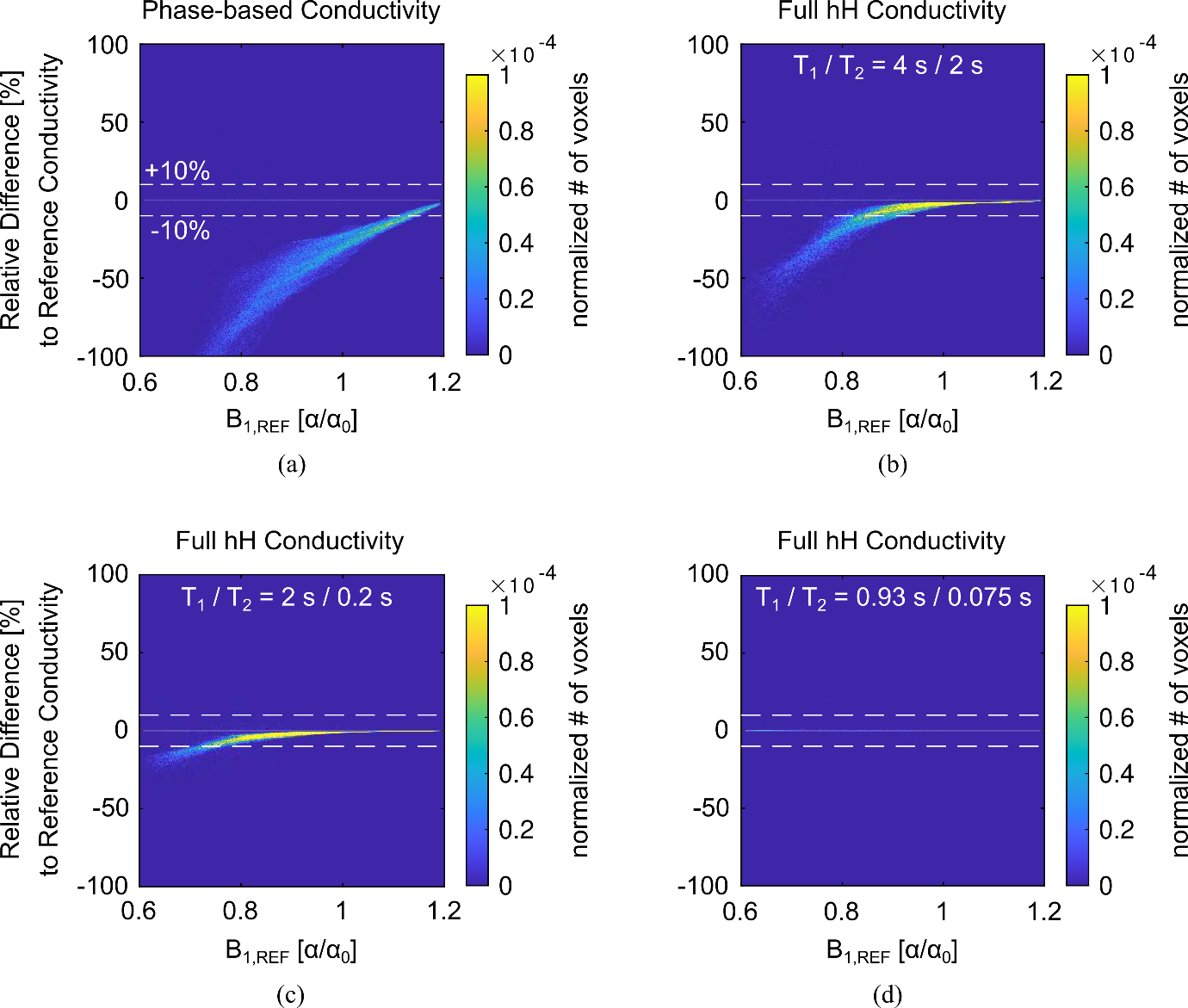


**Supplementary Information Figure S2:** (a) Relative difference between the phase-based and the full hH conductivity based on the reference measurements. (b) Relative difference between the reference conductivity and the full hH conductivity using a simulated B_1_ map of a fluid-like phantom (Case I: T_1_ / T_2_ = 4 s / 2 s). (c) Relative difference between the reference conductivity and the conductivity using a simulated B_1_ map of a phantom with T_1_ / T_2_ = 2 s / 0.2 s (Case II). (d) Relative difference between the reference conductivity and the conductivity using the estimated T_1_ / T_2_ ~ 930 ms / 75 ms of the measured phantom (Case III).

**Turbo Spin Echo (TSE) acquisition**


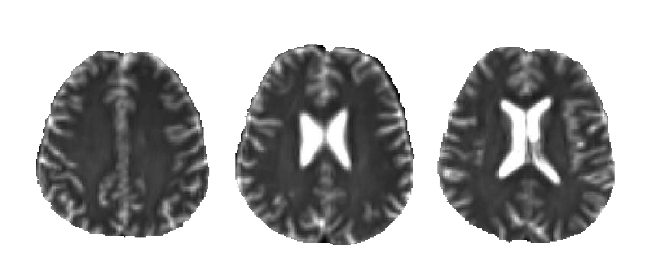


**Supplementary Information Figure S3:** Exemplary axial slices of the TSE image used for the tissue boundary preserving post processing.

**Permittivity Reconstruction**

Similar to the conductivity, the permittivity $\varepsilon$ can be constructed using the full homogeneous Helmholtz (hH) equation as follows [2, 3]:

|  | $\varepsilon= -\frac{1}{\mu_{0}\omega^{2}}Re\left\{ \frac{\nabla^{2}B_{1}^{+}}{B_{1}^{+}} \right\}=-\frac{1}{\mu_{0}\varepsilon_{0}\omega^{2}}\left( \frac{\nabla^{2}B_{1}}{B_{1}}-\left( \nabla\varphi^{+} \right)^{2} \right)$ | [S3] |
| --- | --- | --- |

In the equation, $\varepsilon_{0}$ denotes the vacuum permittivity.

In approximation, the permittivity can also be reconstructed using the magnitude-based reconstruction formula [4, 5]:

|  | $\varepsilon\approx-\frac{1}{\mu_{0}\varepsilon_{0}\omega^{2}}\frac{\nabla^{2}B_{1}}{B_{1}}$ | [S4] |
| --- | --- | --- |


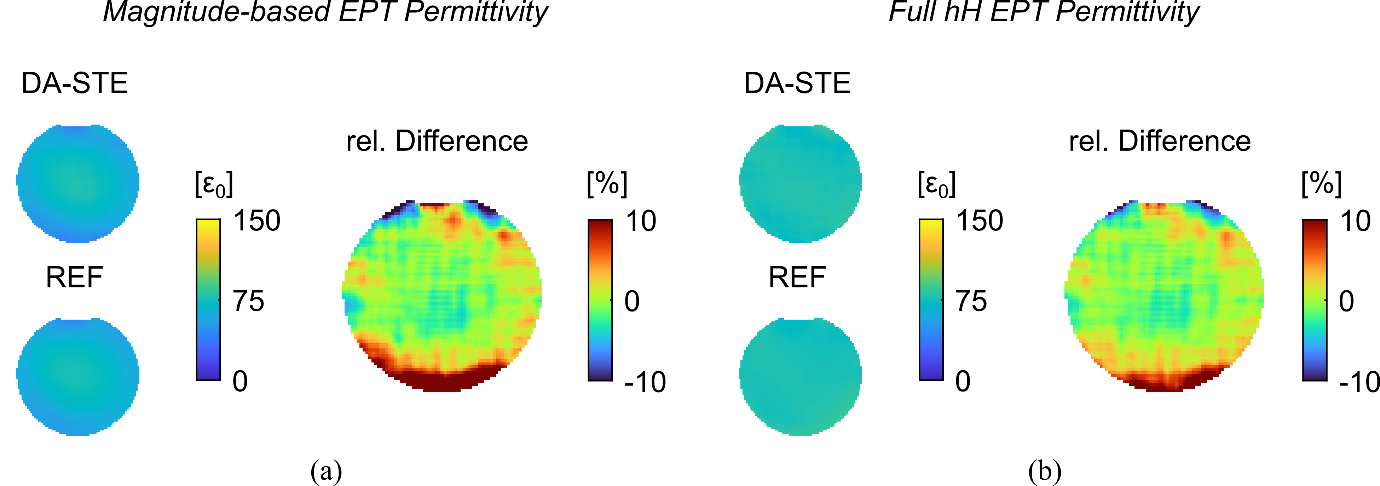


**Supplementary Information Figure S4:** (a) Permittivity maps obtained by magnitude-based EPT for the proposed DA-STE and the reference along with the relative difference map. (b) Permittivity maps obtained by full hH EPT for the proposed DA-STE and the reference and the relative difference map. The expected permittivity for the phantom is 79 ε_0_ [6]. For the reference method, the average permittivity over the whole phantom was found to be (78 ± 3) ε_0_. For DA-STE, the average permittivity is (77 ± 5) ε_0_. Due to outliers at the boundary, voxels within a width of 5 pixels from the edge were excluded.


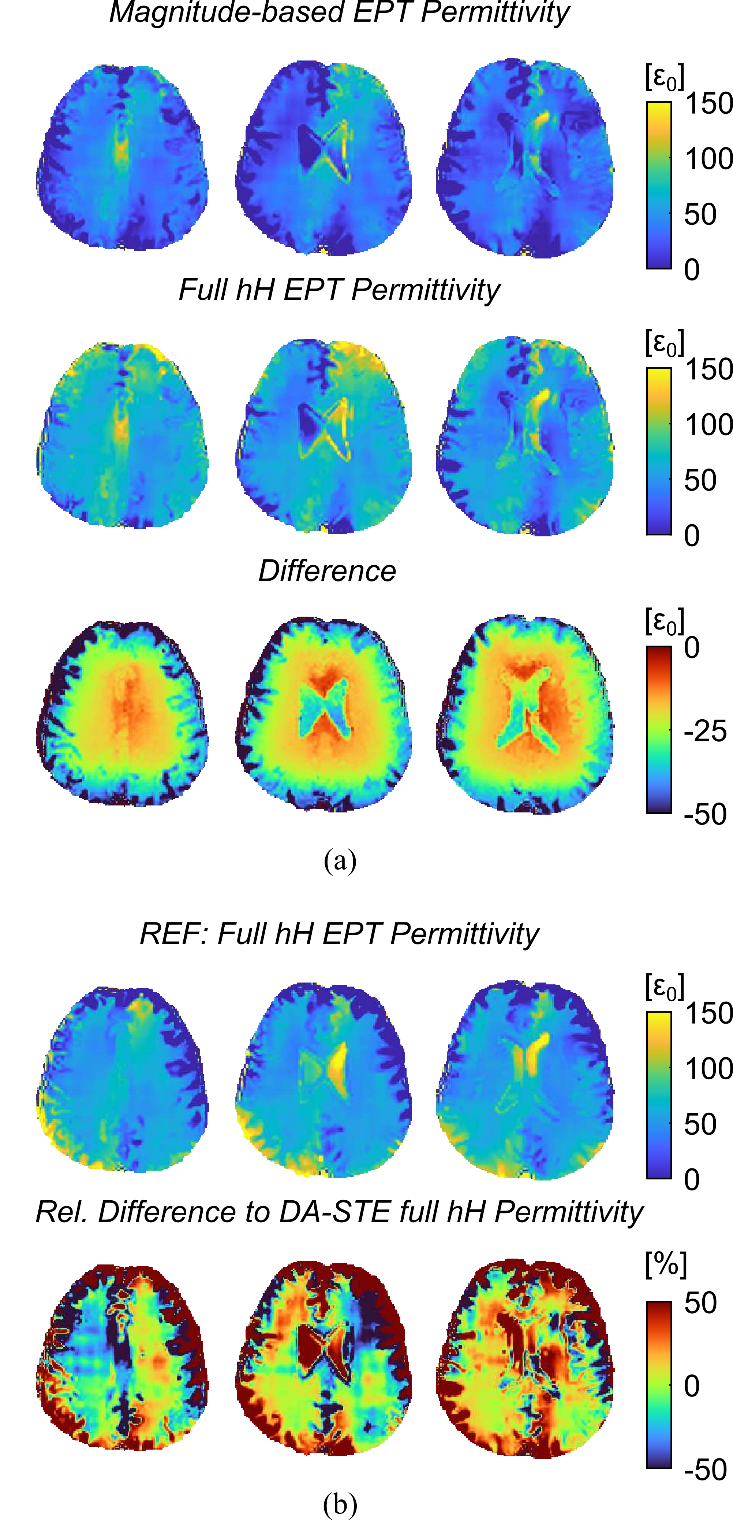


**Supplementary Information Figure S5:** Permittivity reconstructions for the same axial slices shown in Figure 6: (a) the magnitude-based permittivity (top row), full hH permittivity (middle row) and the difference between the two (bottom row). (b) the full hH permittivity of the reference measurements and the relative difference to the full hH DA-STE permittivity.

**References**

1. Pohmann R, Scheffler K (2013) A theoretical and experimental comparison of different techniques for B1 mapping at very high fields. NMR in Biomedicine 26:265–275. https://doi.org/10.1002/nbm.2844

2. Katscher U, Berg CAT van den (2017) Electric properties tomography: Biochemical, physical and technical background, evaluation and clinical applications. NMR Biomed 30:e3729. https://doi.org/10.1002/nbm.3729

3. Katscher U, Kim D-H, Seo JK (2013) Recent Progress and Future Challenges in MR Electric Properties Tomography. Comput Math Method M 2013:. https://doi.org/10.1155/2013/546562

4. Voigt T, Katscher U, Doessel O (2011) Quantitative conductivity and permittivity imaging of the human brain using electric properties tomography. Magn Reson Med 66:456–466. https://doi.org/10.1002/mrm.22832

5. Lier ALHMW van, Raaijmakers A, Voigt T, Lagendijk JJW, Luijten PR, Katscher U, Berg CAT van den (2014) Electrical Properties Tomography in the Human Brain at 1.5, 3, and 7T: A Comparison Study. Magn Reson Med 71:354–363. https://doi.org/10.1002/mrm.24637

6. Stogryn A (1971) Equations for Calculating the Dielectric Constant of Saline Water (Correspondence). IEEE Trans Microw Theory Tech 19:733–736. https://doi.org/10.1109/TMTT.1971.1127617
